# The Effect of Exposure to SARS-CoV-2 Vaccination and Infection on Humoral and Cellular Immunity in a Cohort of Patients with Immune-Mediated Diseases: A Pilot Study

**DOI:** 10.3390/pathogens13060506

**Published:** 2024-06-14

**Authors:** Giulia Anna Maria Luigia Costanzo, Giuseppina Sanna, Francesco Pes, Carla Maria Deiana, Andrea Giovanni Ledda, Andrea Perra, Vanessa Palmas, Valeria Manca, Michela Miglianti, Ferdinando Coghe, Aldo Manzin, Stefano Del Giacco, Luchino Chessa, Davide Firinu

**Affiliations:** 1 Department of Medical Sciences and Public Health, University of Cagliari, 09100 Cagliari, Italy; giuliacostanzo14@gmail.com (G.A.M.L.C.); fra81pes@gmail.com (F.P.); carladeiana95@tiscali.it (C.M.D.); andrea.giovanni.ledda@gmail.com (A.G.L.); m.miglianti420@gmail.com (M.M.); delgiacco@unica.it (S.D.G.); luchinochessa@unica.it (L.C.); davide.firinu@unica.it (D.F.); 2 Microbiology and Virology Unit, Department of Biomedical Sciences, University of Cagliari, 09042 Monserrato, Italy; vanessa.palmas@unica.it (V.P.); mancavaleria26@gmail.com (V.M.); aldomanzin@unica.it (A.M.); 3 Oncology and Molecular Pathology Unit, Department of Biomedical Sciences, University of Cagliari, 09100 Cagliari, Italy; andrea.perra@unica.it; 4 Laboratory Clinical Chemical Analysis and Microbiology, University Hospital of Cagliari, 09042 Monserrato, Italy; fcoghe@aoucagliari.it

**Keywords:** COVID–19, immune-mediated diseases, vaccine, interferon-gamma release assay, infections

## Abstract

Immunization against COVID-19 is needed in patients with immune-mediated inflammatory diseases (IMIDs). However, data on long-term immunity kinetics remain scarce. This study aimed to compare the humoral and cellular response to COVID-19 in patients with immune-mediated inflammatory diseases (IMIDs) compared to healthy controls. We compared the humoral and cellular response to SARS-Cov-2 elicited by vaccination and/or infection in a prospective cohort of 20 IMID patients compared with a group of 21 healthcare workers (HCWs). We assessed immunity before and after the third and fourth dose of BNT162b2 or after COVID-19 infection using quantitative IgG anti-SARS-CoV-2 Spike antibody (anti-S-IgG), neutralization assay, and specific interferon-gamma (IFN-g) release assay (IGRA). The responses were compared with those of healthy controls. The two groups were similar in age and total exposure, becoming infected for the first time, mainly after the third dose. Neutralizing antibodies and IGRA were negative in 9.5% of IMID patients but not in any HCWs. No significant difference was found between neutralization titers to BA.1 in the IMID and the HCW groups. The study highlights the SARS-CoV-2 immunological responses in healthy controls and IMID patients, suggesting that the combined stimuli of vaccination and infection in IMID patients could promote a more profound immunological response.

## 1. Introduction

The COVID-19 pandemic, due to the high mortality and morbidity directly related to the SARS-CoV-2 infection, has attracted the attention of the scientific community over the past three years. Vaccination has already proven to be the most effective measure against spreading the infection and reducing its severity. The SARS-CoV-2 spike protein (S-protein), a large class I trimeric fusion protein, is encoded by a lipid nanoparticle-formulated mRNA vaccine used in the Pfizer-BioNTech COVID-19 (BNT162b2) vaccine.

The vaccine was licensed in Europe and the United States in late 2020. Data on immune responses to BNT162b2 against the original strain have been well highlighted by [1] and demonstrated how the vaccine induces long-lasting SARS-CoV-2 specific spike-protein (and its RBD) specific B-cells and neutralizing antibodies as well as polyspecific CD8+ and CD4+ T-cell clones [2].

The profile of the immune response of the BNT162b2 vaccine remains to be investigated beyond the short term, especially in patients affected by immune-mediated inflammatory diseases (IMIDs) [3], which rendered them vulnerable and thus inserted them in the frail category [4,5].

Among individuals with IMIDs, infections significantly contribute to morbidity and death [6,7] due to the immunosuppressive effects of drugs and the autoimmune illness itself; IMID patients also have an increased risk of infection [6] and a higher risk of hospitalization as a result of COVID-19.

Furthermore, disease-modifying immunosuppressive agents or antirheumatic drugs (conventional, targeted synthetic, or biological) may interfere with vaccination in this population by modulating or suppressing important immune system effectors, which can reduce immunogenicity and vaccine efficacy [7,8]. Therefore, since patients with IMIDs are among frail subjects, vaccine booster doses have been recommended to achieve adequate protection for this susceptible group according to “waning immunity” and the immune escape of variants of concern (VOCs) that emerged over time [9].

The cellular response to SARS-CoV-2 is one of the critical determinants of protection by severe disease [10], as memory T cells can contribute to protection upon viral exposure. T cells have also been shown to be less affected by VOCs’ ability to overcome the protective effect of neutralizing antibodies produced by natural infection and/or vaccination [11]. On the other hand, there is a lack of information on real-world cohorts of vaccinated individuals regarding the COVID-19 mRNA vaccine response in patients with IMIDs beyond the first three doses. After the spread of the omicron strain, the latter phase was the most relevant period for breakthrough infection in many countries.

Breakthrough infections and the resulting hybrid immunity may continue shaping the immune response to SARS-CoV-2, enhancing protection in healthy and immunocompromised subjects [10,12].

To investigate the kinetics of immunity against SARS-CoV-2 in a cohort of IMID patients, we evaluated the cellular and humoral response to SARS-CoV-2 elicited by vaccination and/or subsequent infection as part of prospective observational research (CORIMUN study) throughout the pandemic until early 2023.

## 2. Materials and Methods

### 2.1. Study Design

**Figure 1:**
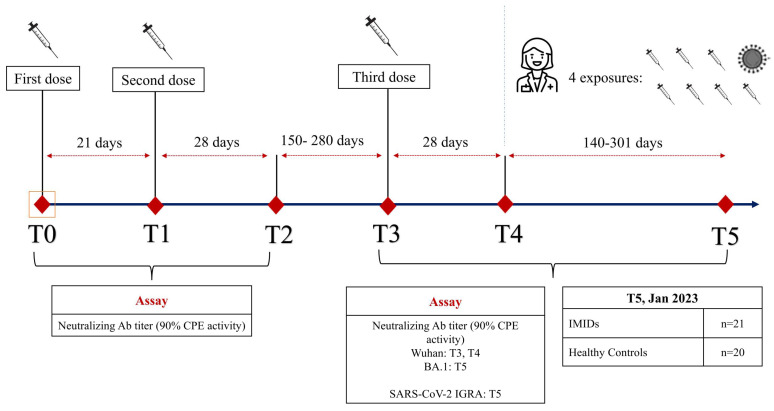
Study design. Legend: IMIDs: immune-mediated inflammatory diseases; Neutralizing ab: serum neutralizing antibody titer assessed by SARS-CoV-2 microneutralization assay (90% protective activity of neutralizing Ab against the CPE induced by the virus); IGRA: Interferon-gamma (IFN-g) release assay to SARS-CoV-2 Spike protein (Wuhan/Hu-1/2019 and 20J/501Y.V3 “gamma” variant).

Humoral and cellular immunity were assessed using quantitative IgG anti-SARS-CoV-2 Spike antibody (anti-S-IgG) and neutralization assay, and specific interferon-gamma (IFN-g) release assay (IGRA) before and after the third dose of BNT162b2, to investigate the responses in a prospective cohort of IMID patients (Figure 1). In addition, we also measured the humoral and cellular immunity after SARS-CoV-2 exposure, which occurred after the completion of a three-dose mRNA vaccine schedule, considering “exposure” to be the fourth vaccine shot and/or infection. The responses were compared with those of a group of healthy controls. 

### 2.2. Patient Enrolment

We prospectively enrolled consecutive adult subjects with Systemic Lupus Erythematosus or Sjögren Syndrome and type 1 Autoimmune Hepatitis who had received the COVID-19 vaccine Pfizer/BioNTech BNT162b2 (mRNA vaccine Comirnaty by Pfizer Inc., New York, NY, USA) and deliberately given their informed consent to participate in the study.

The number and timing of SARS-CoV-2 infections, and demographic and clinical information were collected during a period of 7 months. A control group of subjects was enrolled among healthy healthcare workers (HCWs) consecutively recruited during the same period at our hospital, with no evidence of immunodeficiency or relevant medication intake. The IMID and HCW groups are a sub-cohort of a previous study [13].

We have also reviewed data of serological testing for health surveillance in HCWs for both IgM and IgG antibodies using the 2019-nCov (Snibe, Shenzhen, China) chemiluminescent analytical system (CLIA) assay on the MAGLUMI platform, which detects antibodies from natural infection to SARS-CoV-2 Spike-(S) protein and N-protein with high sensitivity and specificity [14].

At the time of the third mRNA vaccine dose, IMID patients and HCWs were still naïve to SARS-CoV-2 infection. At the next timepoint of the study, patients and controls were enrolled either after a booster dose or after a SARS-CoV-2 infection.

### 2.3. Sample Collection and Storing

Ten mL of peripheral blood was collected by venipuncture. The serum was separated by centrifugation (2000× *g* for 15 min) within 3 h of collection, and aliquots were stored at −80 °C until use.

### 2.4. Outcomes

The outcomes were the seroconversion rate and the presence of neutralizing Abs at the assessment of the residual response of the whole blood SARS-CoV-2 IGRA test.

### 2.5. SARS-CoV-2 Microneutralization Assay (MNA)

MNA was carried out in a Biosafety Level 3 (BSL-3) laboratory (Section of Microbiology and Virology, Cittadella Universitaria di Monserrato) as previously described [15]. Briefly, serum samples were diluted (1:2; 1:5, 1:10,1:40; 1:160; 1:640) in triplicate and mixed with 100 TCID_50_ of SARS-CoV-2 virus (clinical isolate, strain VR PV10734, kindly donated by the Lazzaro Spallanzani Hospital of Rome, Italy) at 37 °C, and serum/virus mixes were added to 96-wells plates containing 5 × 10^5^/mL adherent Vero E6 (ATCC, Manassas, VA, USA) cells which had been seeded the day before. Monolayers were incubated at 37 °C for 72 h before CPE was assessed microscopically and then fixed and stained with Gram’s crystal violet solution. The neutralization percentage of each dilution was calculated by setting the mean OD_595_ of the serum control equal to 100%. The viral dilution used for infection was titrated in each experiment. Cell growth and serum controls were included in each experiment. The highest serum dilution capable of protecting 90% of the infected wells determined the neutralization titers of the serum samples.

### 2.6. SARS-CoV-2 Specific Cellular Immunity

We investigated cell-mediated immunity by measuring IFN-g secreted by T cells in response to SARS-CoV-2 antigens, using a specific IGRA kit with enzyme-linked immunosorbent assay (ELISA) (Covi-FERON ELISA, SD Biosensor, Suwon, Republic of Korea). Whole blood specimens from the participants were collected, and 1 mL was injected into each Covi-FERON tube (Nil tube, SARS-CoV-2 spike protein antigen (Sp)1 tube, Sp2 tube, and Mitogen tube). The Sp1 tube contained spike protein antigens derived from the original SARS-CoV-2 Wuhan/Hu-1/2019 and 20I/501Y.V1 variant, while the Sp2 tube contained those derived from the B.1.351 (20H/501.V2) and P.1 (20J/501Y.V3) variants. After incubation at 37 °C for 16–24 h, plasma was collected by tubes centrifugation at 2200–2300× *g* for 15 min. IFN-g was detected using ELISA, and the measured optical density was converted to IFN-g concentration (IU/mL) using ELISA Report Software (SD Biosensor 2.0, Suwon, Republic of Korea). According to the manufacturer, the positive cut-off for the S and N tubes minus that of the Nil tube was >0.25 IU/mL.

### 2.7. Statistical Analysis

Patient characteristics were summarized using appropriate means, medians, standard deviations, ranges, and percentages. Chi-squared and Fischer’s exact tests were used for categorical data. Mann–Whitney U and Kruskal–Wallis tests were used for unpaired continuous data not normally distributed, and nonparametric Spearman’s rank was used for the correlation test. Linear or logistic regression was used to evaluate the relationship between the dependent variables (e.g., antibody titer and responder status) and patients’ clinical and demographic characteristics as independent variables. All reported p-values represent 2-tailed tests, with *p* ≤ 0.05 considered statistically significant. All variables were analyzed using SPSS Statistics for Windows, version 23.0 (SPSS Inc., Chicago, IL, USA).

### 2.8. Ethical Aspects

Patients were recruited and enrolled in the study protocol at the University Hospital of Cagliari. Written informed consent was obtained from all patients and controls by the local human research committee’s ethical standards (institutional and national). The study protocol, including informed consent procedures, conformed to the ethical guidelines of the Declaration of Helsinki and was approved by the Ethics Committee of the Cagliari University Hospital (27 May 2020; protocol number GT/2020/10894 and extension of 27 January 2021). Records of written informed consent are kept in patients’ files.

## 3. Results

A total of 20 individuals in the HCW group (11 female) and 21 IMID patients (14 female) (*p* = 0.44) were enrolled. The median age of HCWs and IMID was 45 years (IQR 23.6) and 51 (IQR 20.2), respectively (*p* = 0.02). The median number of days since the last event was 301 in the IMID group and 141 in the HCW group. There was a higher median age and a longer time since the last event in the IMID group. The main demographic and clinical details of the enrolled subjects are shown in Table 1.

### 3.1. Humoral Response

The median total exposure (vaccine shots and/or infection) was four events (*p* = 0.21). Due to the study design and local evolution of the pandemic, the infection occurred after the first course of three doses, mostly in the omicron wave. As shown in Table 1, the duration of symptoms and swab positivity were similar; 1 out of 10 infected patients in the IMID group required hospitalization for COVID-19 (*p* = 0.32). During the course of the study in early 2023 (T5), IMID patients and HCWs were sampled for IGRA response after a median of 301 and 141 days after the last vaccine dose or infection, respectively.

There was no significant difference in neutralization titers against the omicron BA.1 variant between the IMID and HCW groups (IMID 1:40, IQR 120; HCWs 1:40, IQR 150; *p* = 0.43).

At the beginning of 2023, the rate of double positivity for nAbs and IGRA was 43% for IMID patients and 90% for HCWs (*p* = 0.001), respectively. At this time point, 43% of IMID patients and 10% of HCWs (*p* = 0.017) resulted positive for neutralizing antibodies, while the rate of both IMID patients and HCWs resulting simultaneously negative for both neutralizing antibodies and IGRA was 9.5% for IMID patients and 0% for HCWs (*p* = 0.157), respectively (Table 2).

A logistic regression was performed to ascertain the effects of different combinations of variables such as age, gender, group, hybrid immunity, ongoing immunosuppressants, and time since last exposure on the likelihood that participants had a positive IGRA test at T5. The logistic regression model resulted in statistically significant χ^2^(4) = 19.19, *p* = 0.0007. The fitted regression model was as follows: IGRA positive = 3154 − 2318 (if IMID) + 1088 (if female)—0.05319 × Age + 2605 (if hybrid). Time from the last event to the examination did not significantly add to the model in any combination.

### 3.2. Quantitative IGRA Response

The magnitude of residual IGRA response to both original and variant S protein in early 2023 was significantly lower in the IMID group than in HCWs. Among IMID patients, the IGRA response to OSP was 0.47 (IQR 0.33) vs. 1.18 (IQR 2.47) among HCWs (*p* = 0.024).

Similarly, the measured response to VSP was 0.44 (IQR 0.71) among IMID patients vs. 1.14 (IQR 2.58) among HCWs (*p* = 0.03).

The HCW group showed no significant difference according to the accrual number of events (vaccine and/or infection) and the magnitude of IGRA response to the original and variant Spike (*p* = 0.33).

Also, in IMID patients, there was no statistically significant positive correlation between the number of events occurring and the magnitude of IGRA response to the original Spike (*p* = 0.92) and Variant Spike (*p* = 0.86).

In the IMID group, we did not find a significant correlation between the magnitude of IGRA response to OSP or VSP and neutralizing antibody titers (*p* = 0.08, rs 0.694 and *p* = 0.07, rs 0.64, respectively), even including those with negative tests.

In the HCW group, we did not find a significant correlation between the magnitude of IGRA response to OSP and VSP and neutralizing antibody titers (*p* = 0.32; rs −0.263 and *p* = 0.69, rs −0.11, respectively).

We did not find a substantial difference in neutralizing antibody titers between IMID patients with hybrid immunity compared to those without it (*p* = 0.81), and similar results were found when comparing HCWs with hybrid immunity to those without (*p* = 0.57).

## 4. Discussion

Our pilot study analyzed the immunological response of IMID patients to SARS-CoV-2 over time after an accrual exposure generated by vaccine shots and infection. We found that the breakthrough exposure to SARS-CoV-2 (or a fourth dose of vaccine) could elicit measurable residual immunological response months later. Therefore, even IMID patients who may have had an impaired response to the vaccine after the three-course vaccine schedule seem to achieve a long-lasting (>6 months) response after a booster dose or after de novo infection. An extraordinary immunization campaign and innovative use of vaccine technology, such as those employing mRNA, particularly in frail patients and those at risk of severe outcomes, limited the impact of the COVID-19 pandemic. A previous study by our group evaluated the antibody response to the BNT162b2 vaccine in a cohort of IMID patients, highlighting that among IMID patients, the vast majority (94%) were responders one month after the second dose [13]. It has already been highlighted that IMID patients showed a lower rate of seroconversion, which underlines the importance of a scheduled vaccination [8].

Subsequently, booster vaccine doses were recommended to provide adequate protection for this susceptible group, following “waning immunity” and the immune escape of VOCs that had emerged over time [9]. Cellular response to SARS-CoV-2 is one of the critical determinants of severe disease protection, especially months after viral and/or vaccine exposure in the context of declining or absent humoral immunity [10]. In addition to antibodies and memory B cells, memory T cells can contribute to protection upon exposure to the virus. T cells have also been shown to be less affected by VOCs’ ability to overcome the protective effect of neutralizing antibodies produced by natural infection and/or vaccination [11].

Previous studies have demonstrated the usefulness of the booster’s strategy in immunocompromised patients. However, there are reports of reduced or absent humoral and cellular responses in patients with IMIDs [16].

The emergence of SARS-CoV-2 variants with increased transmissibility and immune escape potential became a global concern. Several virus variants emerged that escaped neutralization by COVID-19 convalescent and vaccine-induced response and acquired genome mutations similar to those found in variants of concern first identified in the UK, South Africa, and Brazil [17,18,19].

SARS-CoV-2 variants of concern (VOCs) triggered severe endemic waves and vaccine breakthrough infections. The delayed development of alpha S-specific cellular and humoral immunity after VBI suggests reduced immunogenicity against the alpha VOC’s S-protein, while there was a higher and earlier N- and M-reactive T-cell response [20].

Thus, our study aimed to analyze the development of humoral and cellular response and its persistence after repeated exposures to SARS-CoV-2 vaccination or after infection in a small group of patients affected by IMIDs. The humoral response in individuals who are immunosuppressed or undergoing dialysis is typically inadequate, facilitating the long-term presence of the virus and promoting the emergence of viral variants [21].

During the pandemic (mainly during the omicron wave), we sampled subjects who had breakthrough infections or were boosted (fourth dose with the bivalent original/BA.1 vaccine) according to indications for subjects with IMIDs.

The magnitude of IGRA response to both OSP and VSP significantly differed between IMID patients and HCWs. We did not find a significant difference between HCW and IMID antibody titers directed to BA.1.

Breakthrough omicron infections in IMID patients show a high cumulative incidence, with similar rates between patients on immunosuppressants and controls [22].

However, the disease’s severity is usually mild, with additional vaccinations and prior SARS-CoV-2 infections reducing the risk of breakthrough infections [22]. Following omicron breakthrough infection, there is a durable reprogramming of neutralizing antibody responses, with a robust boosting of neutralization activity and an expansion of breadth against other omicron strains [23]. In HCWs, the responses to breakthrough omicron infection occurring after three doses are mainly due to cross-reacting B cells initially induced by vaccination, which generate B-cells that bind both the omicron and Wuhan spike proteins [24]. SARS-CoV-2 vaccines also elicit highly cross-reactive cellular immunity against the omicron variant, providing considerable protection against severe disease [25]. After the third dose, the differences in IFN-gamma response were overall reduced compared to HCWs.

At the beginning of 2023, we found that only 9.5% of IMID patients were double negative for the antibody and IGRA assay after a median of 301 days since the last known exposure to SARS-CoV-2.

Hybrid immunity refers to the combined protection achieved by a combination of natural infection and vaccination. For individuals who have already been vaccinated against SARS-CoV-2 but subsequently become infected with the virus (including a significant number of individuals with immune-mediated inflammatory diseases, primarily infected with omicron variants), the idea of hybrid immunity becomes essential for assessing the level of risk. Newly available data have demonstrated a significant influence of booster shots and hybrid immunity on the responses of immunosuppressed individuals [26].

These data suggest that patients with IMIDs show the development of an immune response after vaccination and/or subsequent infection, which is especially significant when considering a “layered defense” concept [27].

Although not completely, most patients exhibited the primary components of circulating (or tissue) adaptive immunity. Other authors found similar results; our study confirms them in a longer time frame [28]. The improving clinical outcomes of COVID-19 in IMIDs observed during the pandemic may also be related to the fact that our data come from the frequent cases of those who received a three-vaccine regimen and then had SARS-CoV-2 infection [29]. We recognize that our study has limitations. First, the cohort analyzed is small when compared to most clinical cohorts. Other limitations include the monocentric and non-randomized design, the lack of flow-cytometric analysis, and the memory B-cell recall response dissection to SARS-CoV-2. However, the accurate assessment of the humoral immune response, including neutralization with reference assays, together with the T-cell response in terms of specific IGRA, strengthens the validity of our study.

Indeed, the use of live SARS-CoV-2 virus in the neutralization assays, rather than a surrogate model, further strengthens this study. The 90% neutralization titer endpoint used in our study is stricter than the 50% commonly reported in most published studies, as are low (PRNT50)- and high (PRNT90)-stringency plaque reduction neutralization test (PRNT) Ab determinations [14].

In addition, these methods are highly reproducible for detecting SARS-CoV-2-specific responses.

## 5. Conclusions

Our pilot data suggest that after priming with three doses of the mRNA vaccine, infection with omicron (or the second booster) can further increase the proportion of IMID patients with a measurable response months after the last event, as well as the magnitude and the duration of specific antiviral immunity. We may infer that the combined stimuli of vaccination and infection in IMID patients could promote a more profound immunological response. This may promote robust protection against future exposure to SARS-CoV-2 or severe outcomes and should be further assessed in specific experimental and clinical settings.

## Figures and Tables

**Table 1:** The main characteristics of enrolled subjects.

Subject Characteristics	IMIDs n = 20	HCWs n = 21	*p*-Value
Age (years) median (IQR)	51 (20.2)	45 (23.6)	0.02
Female %	67%	55%	0.44
AZA or immunosuppression ongoing, %	94%	0%	<0.001
Median dose of daily oral prednisone	5 mg	0	n.a.
Total events (vaccine and/or infection), median (IQR)	4 (1.6)	4 (0.8)	0.21
Infection after 3rd dose	10.5%	63%	0.0008
Infection after 4th dose	23.5%	0%	0.13
Last event type before sampling at T5	25% infection 75% vaccine	47% infection 52% vaccine	0.21
Days since the last event before sampling at T5 (IQR)	301	141	0.043
Hospital admission for COVID-19	1/10	0/16	0.32
Duration of swab positivity, median (IQR)	7 (12.7)	7 (10.5)	0.75
Duration of COVID-19 symptoms, median (IQR)	1 (6.3)	4 (4.5)	0.57

**Table 2:** Categorization of humoral and cellular response after SARS-CoV-2 infection or booster at T5.

	Residual Response after Additional Exposures (SARS-CoV-2 Infection or Booster), in January 2023 (T5)								
	# Of Exposures	Last Event to Sampling (Days)	nAb Titer [1:X] BA.1	IGRA OSP IU/mL	IGRA VSP IU/mL	Rate of Residual Responders IGRA	Double Positive to nAb and IGRA	Rate of Isolated nAb Positivity	Rate of Double Negative to nAb and IGRA
IMID group	4 (1.6)	(301)	40 (0)	0.47 (0.33)	0.44 (0.71)	43%	43%	43%	9.5%
HC group	4 (0.8)	(141)	40 (150)	1.18 (2.74)	1.14 (2.58)	90%	90%	10%	0%
*p* value	0.21	0.043	0.43	0.024	0.03	0.0015	0.001	0.017	0.157

## Data Availability

Data are available to the corresponding author upon reasonable request.

## Abbreviations

IMIDs: immune-mediated inflammatory diseases; HCWs: healthcare workers; IGRA: specific interferon-gamma (IFN-g) release assay; VOC: variants of concern.
